# Preparation, Optimization, and Characterization of Bovine Bone Gelatin/Sodium Carboxymethyl Cellulose Nanoemulsion Containing Thymol

**DOI:** 10.3390/foods13101506

**Published:** 2024-05-13

**Authors:** Mengying Liu, Ruheng Shen, Liyuan Wang, Xue Yang, Li Zhang, Xiaotong Ma, Long He, Aixia Li, Xiangying Kong, Hongmei Shi

**Affiliations:** 1 College of Food Science and Engineering, Gansu Agriculture University, Lanzhou 730070, China; liumengying1860@163.com (M.L.); ruhengshen@163.com (R.S.); wly04122022@163.com (L.W.); yangxuelyh@163.com (X.Y.); maxiaotong17@163.com (X.M.); hel@gsau.edu.cn (L.H.); liax@gsau.edu.cn (A.L.); 2 Qinghai Haibei Animal Husbandry and Veterinary Science Research Institute, Haibei 810200, China; 13897309088@163.com; 3 Gansu Gannan Animal Husbandry and Veterinary Workstation, Hezuo 746300, China; 18418698062@163.com

**Keywords:** bovine bone gelatin, nanoemulsion, process optimization, structures, antioxidants

## Abstract

The aim of this study is to produce a biodegradable food packaging material that reduces environmental pollution and protects food safety. The effects of total solids content, substrate ratio, polyphenol content, and magnetic stirring time on bovine bone gelatin/sodium carboxymethylcellulose nanoemulsion (BBG/SCMC–NE) were investigated using particle size, PDI, turbidity, rheological properties, and zeta potential as evaluation indexes. The micro, structural, antioxidant, encapsulation, and release properties were characterized after deriving its optimal preparation process. The results showed that the nanoemulsion was optimally prepared with a total solids content of 2%, a substrate ratio of 9:1, a polyphenol content of 0.2%, and a magnetic stirring time of 60 min. SEM showed that the nanoemulsion showed a dense and uniform reticulated structure. FTIR and XRD results showed that covalent cross-linking of proteins and polysaccharides altered the structure of gelatin molecular chains to a more compact form but did not change its semi-crystalline structure. DSC showed that the 9:1 BBG/SCMC–NE had a higher thermal denaturation temperature and greater thermal stability, and its DPPH scavenging rate could reach 79.25% and encapsulation rate up to 90.88%, with excellent slow-release performance. The results of the study provide basic guidance for the preparation of stable active food packaging with excellent properties.

## 1. Introduction

In recent years, food safety has become an important issue affecting people around the world [1]. Food packaging plays a very important role in preventing chemical, physical, and biological contamination during food storage and distribution [2]. Edible biodegradable polymers are receiving increasing attention worldwide due to concerns about environmental protection and food safety [3,4]. Natural edible polymers are widely used to encapsulate bioactive ingredients such as antioxidants and antimicrobials in functional foods, which can control their release and regulate their bioavailability [5,6]. Degradable biopolymer matrices are usually in the following basic classes: proteins, polysaccharides, and lipids [7]. Usually, degradable biopolymers containing a single component lag behind in certain properties, while composites combining two or more components can improve these properties [8]. Moreover, composite biopolymers generally have the ability to block water and oxygen penetration, thereby reducing water and weight loss, preventing the release of flavor substances, slowing oxidation reactions, and inhibiting microbial contamination [9]. Among them, protein–polysaccharide biocomposites can enhance the physical, mechanical, and barrier properties of protein–polysaccharide composites due to hydrophobic–hydrophobic and/or electrostatic interactions in their polymer networks [10]. It is also necessary to impart certain antimicrobial and antioxidant properties to composites by adding active substances [11].

Gelatin is a natural product formed by partial hydrolysis of collagen in connective tissues such as animal skin, bone, and muscle membranes, which has low cost, good oxygen resistance, and film-forming properties, making it widely used in food preservation [12]. Current studies have used skin-derived gelatin to prepare gelatin films/coating, e.g., Hu et al. [12] prepared a corn protein–gelatin composite film containing allicin and completely wrapped chilled beef in the film, which effectively delayed the quality deterioration of beef during cold storage. Du et al. [13] prepared gelatin films doped with titanium dioxide (TiO_2_) nanoparticles, thymol, and β–cyclodextrin, which resulted in good water solubility, tensile strength, and biocompatibility and significantly extended the shelf life of strawberries and tomatoes. In contrast, bovine bone gelatin (BBG) is biodegradable, biocompatible and non–toxic and is rich in natural components such as amino acids [14]; it also has good antioxidant properties [15], which has great potential in the field of food preservation. However, the low water resistance and poor mechanical strength of bovine bone gelatin have severely limited its application [16]. To solve this problem, sodium carboxymethyl cellulose (SCMC), which has good biocompatibility, biodegradability, and a relatively low cost, can be selected to be compounded with it, which can significantly alleviate the disadvantages of gelatin [3]. For example, Lv et al. [17] developed films with good compatibility, tensile strength, and elongation at break and excellent antioxidant and antimicrobial properties using gelatin and sodium carboxymethylcellulose as substrates, which had significant preservation effects on fruits. In addition, Fan et al. [18] showed that protein–polyphenol conjugates have stronger antioxidant activity and emulsification stability than pure proteins. Thymol (THY), as a major component of the essential oils of anise, clove, cinnamon, and thyme, has a high food safety and a wide range of antimicrobial activities [19]. Ding et al. [20] developed an edible antimicrobial coating using konjac glucomannan and low acyl gellan gum as substrates in combination with thymol nanoparticles that significantly extended the shelf life of blueberries to 42 d. However, one of the most common drawbacks of direct binding of polyphenols to proteins/polysaccharides is the rapid migration of polyphenols from the complex, which greatly reduces the complex’s activity [2]. To overcome this disadvantage, one measure is to encapsulate polyphenols in biopolymers, and the most suitable encapsulation technology is nanocapsules/particles/emulsions based on biopolymers [21].

Nanoemulsion (NE) droplets are small and stable with an average particle size of 20–200 nm and are homogeneous dispersion systems formed by dispersing one immiscible solution into another, forming the main nanocarrier systems for encapsulating and delivering active substances [22]. In addition, nanoemulsion offer the advantages of higher transparency, higher stability, better controlled release, better bioactivity, and enhanced physicochemical properties [21]. There have been extensive studies applying nanotechnology to encapsulate actives in biodegradable biopolymers; e.g., Mutlu [2] prepared nanoemulsion loaded with grapefruit essential oils, which were incorporated into gelatin/sodium alginate-based films and significantly improved the physical, mechanical, and antimicrobial properties of the films. Liu et al. [23] prepared nanoemulsion loaded with carvacrol by a high-energy shear method, which solved the volatility and instability of carvacrol during use and made its antimicrobial effect more effective and long-lasting. And there are often other issues that need to be addressed when making nanoemulsion. For example, many encapsulated bioactive components degrade when exposed to elevated levels of light, oxygen, or temperature, thereby reducing their activity. In such cases, optimization of composition, handling, etc., is often required to maintain good physical and chemical stability of the nanoemulsion [24]. For improving the functional properties of nanoemulsion, physical techniques such as ultrahigh pressure and ultrasound are often used. Among them, nanoemulsion prepared by ultrasonic have smaller droplet sizes, lower polydispersity index (PDI), and higher stability [25]. In addition, cavitation generated by ultrasound can further expand the tertiary structure of gelatin, which can promote the interaction between gelatin and polysaccharides and polyphenols [26].

The aim of this study was to optimize BBG/SCMC nanoemulsion encapsulating THY and to determine the effect of total solids content, substrate ratio, actives concentration, and magnetic stirring time on the physicochemical properties of BBG/SCMC nanoemulsion. In addition, the present study challenged the adoption of edible biopolymers as an innovative carrier for the delivery of THY. A protein–polysaccharide complexation was facilitated by Ca^2+^–induced cross–linking. The composites were then rigorously analyzed using a suite of techniques to assess patterns of change in their microscopic, structural, and thermal stability as well as oxidation resistance, encapsulation rate, and release properties.

## 2. Materials and Methods

### 2.1. Materials

Beef bone gelatin (BBG, Frozen Force 120) was obtained from Zhenghong Biotechnology Co., Ltd. (Guangzhou, China). Sodium carboxymethyl cellulose (SCMC) was acquired from Shanghai Changguang Enterprise Development Co., Ltd. (Jinshan District, Shanghai, China). Fresh beef chops and food-grade peanut oil are obtained from local supermarkets. Thymol (THY) was acquired from Shanghai McLean Biochemical Technology Co., Ltd. (Shanghai, China). Food-grade calcium chloride (CaCl_2_) was used as a cross-linking agent for the nanoemulsion (O/W) (Jiangsu Kolondo Food Ingredients Co., Ltd., Lianyungang City, China). Food-grade Tween 80 as surfactant for the nanoemulsion (Guangdong Runhua Chemical Co., Ltd. Meide City, China).

### 2.2. Preparation of BBG/SCMC Emulsion

**Figure 1:**
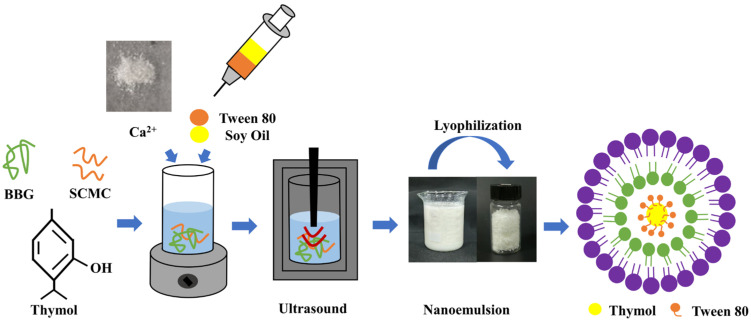
Preparation of bovine bone gelatin/sodium carboxymethyl cellulose nanoemulsion loaded with thymol by ultrasonic technique.

A schematic diagram of the preparation of BBG/SCMC by ultrasonic technique is shown in Figure 1. Firstly, a certain amount of BBG and SCMC were weighed and dissolved in distilled water at room temperature for 10 min and then stirred uniformly at 60 °C and 800 rpm for 60 min, respectively. Thereafter, the solution was cooled to room temperature to allow sufficient hydration and then mixed to obtain the aqueous phase. The oil phase was prepared by dissolving thymol in 2% (*w*/*v*) peanut oil and 2% (*w*/*v*) Tween 80. Finally, a certain proportion of food-grade calcium chloride and the oil phase were slowly added to the aqueous phase, and the crude emulsion was obtained by magnetic stirring at 1200 rpm for 10 min (BBG/SCMC–CE). The crude emulsion was subjected to an ultrasonic bath for 10 min to eliminate air bubbles before being placed in an ultrasonic cell crusher to sonicate it for 10 min (ultrasonic on and off time of 5 s and power of 500 W, respectively) to obtain the nanoemulsion (BBG/SCMC–NE). The obtained nanoemulsion were freeze-vacuum dried for 48 h and stored at 4 °C away from light for subsequent process optimization. Based on the optimization test, the microstructure, antioxidant, and thermal stability as well as encapsulation and release properties of the nanoemulsion were further analyzed and determined under different substrate ratios.

### 2.3. Process Optimization of Bovine Bone Gelatin-Based Nanoemulsion

#### 2.3.1. One-Factor Experiments

The total solids content (BBG and SCMC) of the prepared nanoemulsion was selected to be 1, 2, 3, 4, and 5% (*w*/*v*); polyphenol content to be 0.1, 0.2, 0.3, 0.4, and 0.5% (*w*/*v*); the ratio of bovine bone gelatin to sodium carboxymethyl cellulose to be 10:0, 9:1, 8:2, 7:3, 6:4, and 5:5; and the magnetic stirring time to be 30, 40, 50, 60, and 70 min. Average emulsion particle size, PDI dispersion index, turbidity, rheological characteristics, and zeta potential were used as indicators to determine optimal process parameters for each factor.

#### 2.3.2. Box–Behnken Design

Based on the one-way experiment, the Puntipa Pongsumpun [25] method was referred and slightly modified. According to the principle of Box–Behnken experimental design, four factors of total solids content (1, 2, and 3%), polyphenol content (0.1, 0.2, and 0.3%), magnetic stirring time (50, 60, and 70 min) at three levels, zeta potential, and encapsulation rate were selected as the response values for optimization. Design–Expert 13.0 software was applied to design the factor levels of the response surface analysis tests, as shown in Table 1. Each test was repeated three times. BBD was performed to investigate the effect of each independent variable and the effect of the interaction between the independent variables. And the second order polynomial equation was applied as the response function of the four independent variables (Equation (1)).

(1) $$Y_{i}=a_{0}+a_{1}A+a_{2}B+a_{3}C+a_{4}D+a_{11}A^{2}+a_{22}B^{2}+a_{33}C^{2}+a_{44}D^{2}+a_{12}AB+a_{13}AC+a_{14}AD+a_{23}BC+a_{24}BD+a_{34}CD$$

where Y_i_ is the response function (i = 1, 2, 3, and 4), *a*_0_ is a constant, and the coefficients in Equation (1) are linear coefficients (*a*_1_, *a*_2_, *a*_3_, and *a*_4_), quadratic coefficients (*a*_11_, *a*_22_, *a*_33_, and *a*_44_), and interaction coefficients (*a*_12_, *a*_13_, *a*_14_, *a*_23_, *a*_24_, and *a*_34_), respectively.

#### 2.3.3. Average Particle Size, Zeta Potential, and Polydispersity Index (PDI)

The average particle size, polydispersity index (PDI), and zeta potential of BBG/SCMC emulsions were measured using a nanoparticle size analyzer (Bettersize2000, Dandong Baxter Instrument Co., Ltd., Dandong City, China), and these parameters characterized the distribution of the oil phase and actives in the emulsions. Prior to measurement, BBG/SCMC emulsions were diluted 100 times with ultrapure water and shaken well to avoid the effects of multiple scattering and droplet size distribution. Each measurement was performed three times at 25 °C, and the data were averaged.

#### 2.3.4. Turbidity

Turbidity was determined with reference to Zhang et al. [27] and Gao et al. [28] with minor modifications. The BBG/SCMC emulsion was diluted 10 times with ultrapure water and measured at 600 nm using a Varioskan Flash Fluorescent Enzyme Marker, and the obtained optical density (OD_6oo_) value was used to indicate turbidity. Ultrapure water was used as a blank. Each test was repeated three times.

#### 2.3.5. Rheological Properties

The rheological properties of BBG/SCMC emulsions were analyzed and determined using a rheometer (Discovery HR–3, Waters Technology Co., Ltd., Shanghai, China). The fixture disc was 1 mm thick and 40 mm wide, the sample was equilibrated for 60 s before the test, 1 mL of the emulsion was sucked onto the test disc, the temperature was set at 25 ± 0.1 °C, the plate spacing was set at 100 µm, and the range of the shear rate was 0.1–100 s^−1^; thus, the apparent viscosity change curves of the emulsions were determined.

### 2.4. Characterization of BBG/SCMC–NE

#### 2.4.1. Scanning Electron Microscope (SEM)

An appropriate amount of BBG/SCMC freeze-dried emulsion powder was taken and attached to the conductive adhesive tape, followed by gold spraying. Microstructural characterization of BBG/SCMC was performed using a scanning electron microscope (JEOL S–3400N, Hitachi, Japan) with an acceleration voltage of 10.0 kV.

#### 2.4.2. Fourier-Transform Infrared (FTIR) Analysis

Fourier-transform infrared (FTIR) spectra of BBG/CMC emulsions were recorded using an FTIR spectrometer (FTIR, Nicolet iS50, Thermo Fisher Scientific, Waltham, MA, USA) with reference to the method of Chavoshi et al. [29]. Dried samples were ground into powder and placed on a disk for testing. A portion of the ground and lyophilized sample was taken and placed on the disk for testing. The scanning range was 4000–400 cm^−1^ with a resolution of 4 cm^−1^, and 16 scans were performed for each sample.

#### 2.4.3. X-ray Diffraction (XRD) Analysis

The crystalline and amorphous structures of freeze-dried nanoemulsion powders were measured using an X-ray diffractometer (XD–6, Beijing Pulse General Instrument Co., Ltd., Beijing, China) with reference to the method of Asdagh et al. [30]. The voltage and current were 35 kV and 25 mA, respectively; the power was 1.5 kW; the scanning range 2θ was set to 5°–50°; and the scanning rate was 8°/min. Appropriate samples were taken on the sample stage, and XRD spectra were determined by lightly pressing a slide into a uniform flat state.

#### 2.4.4. Differential Scanning Calorimetry (DSC) Analysis

The thermal stability of the samples was determined using the method of Zhang et al. [31] using a differential scanning calorimeter (DSC25, Waters Corporation, Milford, MA, USA). First, 2–5 mg of the lyophilized sample was weighed and sealed under pressure in an aluminum pan. The baseline was determined using an empty sealed pot, and the samples were heated from 20 °C to 200 °C at a rate of 10 °C/min under a constant nitrogen purge (20 mL/min).

#### 2.4.5. DPPH Free Radical Scavenging Capacity

The antioxidant activity of BBG/CMC emulsions was determined using the method of Soltanzadeh et al. [6] using DPPH (2,2–diphenyl–1–picrylhydrazyl) radical scavenging activity. The lyophilized sample (10 mg) was vortex-mixed with 3.9 mL of a DPPH–ethanol mixture (0.1 mM) in a centrifuge tube. The resulting solution was then protected from light for 30 min, and the absorbance was determined at 517 nm using a UV–vis spectrophotometer (UV–2550, Shimadzu (Shanghai) Laboratory Equipment Co., Shanghai, China) DPPH (%) was calculated using Equation (2):

(2) $$DPPH=\left(A_{0}-A_{1}\right)/A_{0}$$

where *A*_0_ is the absorbance of ethanol instead of the sample added to the DPPH–ethanol mixture, and *A*_1_ is the absorbance of the sample plus the DPPH–ethanol mixture.

#### 2.4.6. Encapsulation Ratio

The method of Liu and Liu [32] was used to measure the thymol encapsulation rate in BBG/SCMC emulsions by UV–spectrophotometry. Thymol was dissolved in anhydrous ethanol to form a thymol solution with a concentration gradient of 5, 10, 20, 30, 40, 60, 80, and 100 μg/mL. Then, 100 μL of the above thymol solution was taken and mixed with 1.9 mL of anhydrous ethanol, respectively. Absorbance at 275 nm was measured using the UV–2550 UV spectrophotometer. The standard curve was plotted using experimental data to obtain the standard curve equation (y = 0.0111x + 0.1087, R^2^ = 0.9995), where y and x represent the absorbance and concentration of thymol, respectively. Next, 100 μL of freshly prepared BBG/SCMC emulsion was mixed with 4.9 mL of anhydrous ethanol and centrifuged at 10,000 rpm for 5 min at ambient temperature, and its absorbance at 275 nm was measured. The absorbance was then fed into the standard curve equation to calculate the free thymol content. The control (without thymol) was used as blank. The encapsulation rate of thymol in the nanoemulsion was calculated using Equation (3):

(3) $$EE\;(\%)=\left(m_{0}-m_{1}\right)/m_{0}$$

where *m*_0_ indicates the actual thymol content added/mg, and *m*_1_ indicates the free thymol content calculated by substituting the measured value into the standard curve/mg.

#### 2.4.7. Release Properties of Thymol

To analyze the release characteristics of thymol from the BBG/SCMC emulsion at 25 °C, 1 g of BBG/SCMC was suspended in phosphate-buffered saline (PBS) and fixed to 100 mL, the volumetric flasks were placed in a constant-temperature water-bath oscillator (25 °C, 150 r/min) for the reaction, 2 mL of the releasing solution was taken at regular intervals, and the PBS was replenished to maintain the constant volume of the solution. Then, 2 mL of the release solution was fixed to 10 mL with anhydrous ethanol and centrifuged at 8000 r/min for 10 min; then, the absorbance of the release solution was measured at 275 nm, and the released thymol content was calculated by substituting the absorbance into the standard curve obtained from 2.9, and the cumulative thymol release rate was calculated according to Formula (4) [33].

(4) $$Cumulative\;release\;rate\;(\%)=m/m_{0}$$

where *m* is the cumulative release of thymol at different time points/mg; *m*_0_ is the total thymol encapsulation/mg.

### 2.5. Statistical Analysis

All experiments in this study were performed three times, and data are reported as mean ± standard deviation. One-way ANOVA and Tukey’s test (IBM SPSS Statistics 27) were used to compare mean differences at the 5% significance level.

## 3. Results

### 3.1. Effect of Independent Variables on Nanoemulsion

#### 3.1.1. Effect of Different Treatment Conditions on Emulsion Particle Size and PDI Dispersion Index

**Figure 2:**
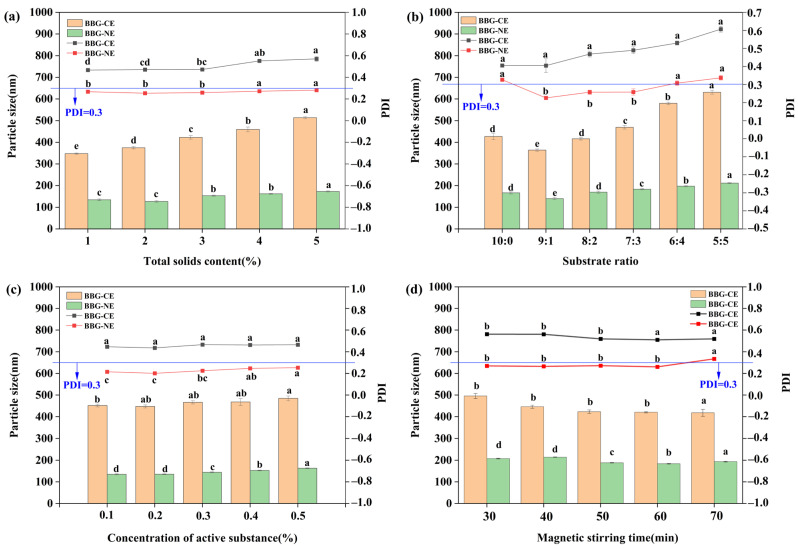
Effect of total solids content on BBG/SCMC–CE and BBG/SCMC–NE particle size and PDI (**a**). Effect of substrate ratio on BBG/SCMC–CE and BBG/SCMC–NE particle size and PDI (**b**). Effect of active concentration on BBG/SCMC–CE and BBG/SCMC–NE particle size and PDI (**c**). Effect of magnetic stirring time on BBG/SCMC–CE and BBG/SCMC–NE particle size and PDI (**d**). Different lowercase letters indicate significant differences between different levels of the factor (*p* < 0.05).

Particle size and PDI dispersion index are one of the key indicators for evaluating the homogeneity of nanoemulsion [34]. As shown in Figure 2a, BBG/SCMC–CE and BBG/SCMC–NE particle sizes tended to increase significantly with increasing total solids content. This is consistent with Tu et al. [35], who reported that alginate–gelatin microcapsules increased in particle size and narrowed the range of particle size distribution with increasing gelatin concentration, which may be due to increased protein concentration, resulting in aggregation and settling due to its incomplete reaction. Meanwhile, BBG/SCMC–CE PDI increased gradually with the increase in total solids content (Figure 2b). The PDI of BBG/SCMC–NE, on the other hand, first decreased and then increased, reaching the lowest at 2% total solids content. Generally, PDI values between 0–0.30 indicate that the emulsion system is uniformly dispersed, and smaller PDI values indicate a more concentrated distribution of particle size and better homogeneity of particle size [36,37]. In addition, the particle size and PDI of BBG/SCMC–CE and BBG/SCMC–NE first decreased and then increased with the decrease in BBG ratio (Figure 2c) (*p* < 0.05). This may be caused by the inability of the polysaccharide quantity to effectively bind protein molecules [38]. Meanwhile, the particle size and PDI of BBG/SCMC–CE and BBG/SCMC–NE gradually increased with the gradual increase in polyphenol content (Figure 2d). The reason for this result may be due to the hydrophobicity of thymol, which opens the α-helical structure of gelatin, and part of the peptide chain structure is unfolded; more internal hydrophobic groups are exposed, resulting in an increase in particle size [34]. The particle size and PDI of BBG/SCMC–CE and BBG/SCMC–NE, on the other hand, decreased gradually with magnetic stirring time and then stabilized. This may be due to the need for sufficient stirring time of the emulsion to obtain small, monodisperse particles and avoid the formation of particle aggregates [39]. In addition, the shear force generated by ultrasonic cavitation helps to reduce the particle size of proteins by disrupting hydrogen bonding as well as hydrophobic and electrostatic interactions so that the particle size of nanoemulsion is significantly lower than that of coarse emulsions (*p* < 0.05) [40]. Tao et al. [41] also noted that the smaller droplet size of emulsions after sonication contributes to the stability of emulsions.

#### 3.1.2. Effect of Different Treatment Conditions on the Turbidity of Emulsions

**Figure 3:**
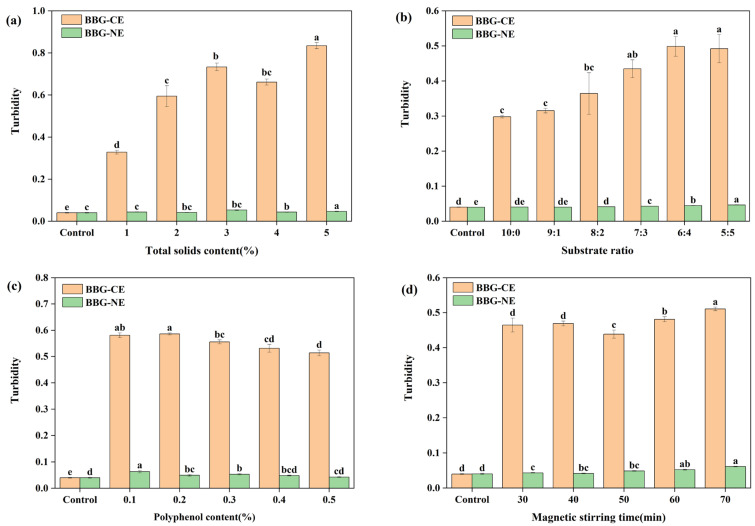
Effect of total solids content on turbidity of BBG/SCMC–CE and BBG/SCMC–NE (**a**). Effect of substrate ratio on turbidity of BBG/SCMC–CE and BBG/SCMC–NE (**b**). Effect of active concentration on turbidity of BBG/SCMC–CE and BBG/SCMC–NE (**c**). Effect of magnetic stirring time on turbidity of BBG/SCMC–CE and BBG/SCMC–NE (**d**). Different lowercase letters indicate significant differences between different levels of the factor (*p* < 0.05).

Turbidity measurements are an indirect method of characterizing the stability of nanoemulsion, with lower turbidity indicating higher solubility [42]. Figure 3a shows that with the gradual increase in total solids content, BBG/SCMC–CE turbidity first increased and then decreased before continuing to increase, and BBG/SCMC–NE turbidity showed a significant upward trend (Figure 3b) (*p* < 0.05). And with the BBG ratio decreasing, the turbidity of both BBG/SCMC–CE and BBG/SCMC–NE showed a significant upward trend (Figure 3c) (*p* < 0.05). This may be due to the fact that the decrease in protein percentage promotes the unfolding of BBG structure, which exposes hydrophobic groups and enhances protein–polysaccharide interactions, leading to protein–polysaccharide aggregation, which results in turbid emulsions and reduced solubility [43]. The turbidity of BBG/SCMC–CE showed a tendency to first increase and then gradually decrease with the increase in polyphenol content, while the turbidity of BBG/SCMC–NE gradually decreased. This may be due to the presence of polyphenols in the emulsion structure, which led to a significant increase in protein solubility [44]. The opposite results were observed for magnetic stirring time and polyphenol content (Figure 3d). BBG/SCMC–CE decreased and then gradually increased over time, whereas BBG/CMC–NE showed a gradual decrease in turbidity, which may be due to the increase in turbidity caused by the formation of more and larger aggregates in the heated protein solution [45]. These results have important implications for further analysis and study of protein (BBG)–polysaccharide (SCMC) complex interactions as encapsulated active ingredients [46].

#### 3.1.3. Effect of Different Treatment Conditions on Rheological Properties of Emulsions

**Figure 4:**
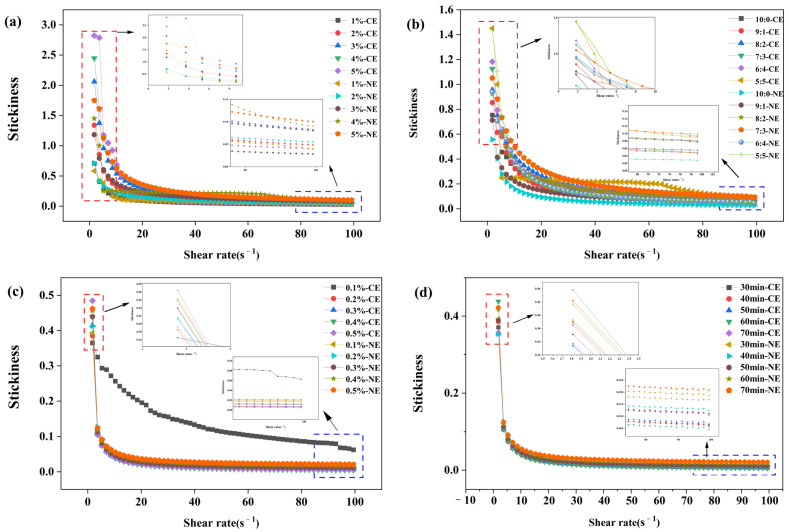
Effect of total solids content on rheological properties of BBG/SCMC–CE and BBG/SCMC–NE (**a**). Effect of substrate ratio on rheological properties of BBG/SCMC–CE and BBG/SCMC–NE (**b**). Effect of active concentration on rheological properties of BBG/SCMC–CE and BBG/SCMC–NE (**c**). Effect of magnetic stirring time on rheological properties of BBG/SCMC–CE and BBG/SCMC–NE (**d**).

Figure 4 shows the dependence of apparent viscosity on shear rate. The apparent shear viscosity of both emulsions decreased with increasing shear rate. The apparent viscosities of BBG/SCMC–CE and BBG/SCMC–NE decreased from 238.03 and 204.00 Pa·s to 0.04–0.46 Pa·s when the shear rate was increased to 20 s^−1^, during which non–Newtonian fluid shear thinning occurred [47]. This can be explained by a reduction in physical interactions between adjacent polymer chains or by structural breakdown [48]. And the apparent viscosities of BBG/SCMC–CE and BBG/SCMC–NE showed a gradual increase (*p* < 0.05) with the increase in total solids content, BBG ratio, polyphenol content, and the extension of magnetic stirring time (Figure 4a–d). This may be due to the formation of an extended network in the aqueous phase of polysaccharides, which can enhance emulsion viscosity and improve emulsion stability [49]. In addition, at high shear rates, the inhomogeneous structure of polysaccharides becomes ordered and less resistant to the flow direction, and thus, the viscosity of the entire emulsion decreases [50]. The high viscosity of the continuous phase is a key factor for emulsion stabilization, as it prevents oil droplet aggregation. However, excessive viscosity also impairs emulsion fluidity and sensory quality [51]. It can be concluded that the blending of polysaccharides and proteins, the addition of polyphenols, and the prolongation of magnetic stirring time can effectively improve the rheological properties of emulsions, increase the apparent viscosity of BBG/SCMC–NE, and improve the stability of emulsions and nanoemulsions.

#### 3.1.4. Effect of Different Treatment Conditions on Zeta Potential of Emulsion

**Figure 5:**
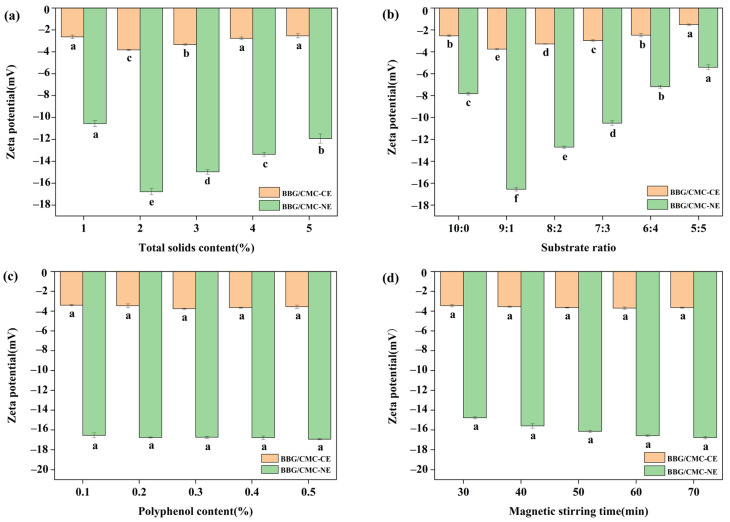
Effect of total solids content on BBG/SCMC–CE and BBG/SCMC–NE zeta potential (**a**). Effect of substrate ratio on BBG/SCMC–CE and BBG/SCMC–NE zeta potential (**b**). Effect of active concentration on BBG/SCMC–CE and BBG/SCMC–NE zeta potential (**c**). Effect of magnetic stirring time on BBG/SCMC–CE and BBG/SCMC–NE zeta potential (**d**). Different lowercase letters indicate significant differences between different levels of the factor (*p* < 0.05).

Zeta potential is a measure of the magnitude of electrostatic repulsion/attraction between particles and an important parameter reflecting the physicochemical stability of nanoparticles under storage conditions [52]. As shown in Figure 5a, the zeta potential of BBG/SCMC–CE and BBG/SCMC–NE first increased and then gradually decreased with increasing total solids content (*p* < 0.05), with the highest zeta potential reaching −16.78 ± 0.48 mV at a total solids content of 2%. Zeta potential is a key indicator of the stability of nanoemulsion, and the higher the absolute value of the zeta potential, the more stable the emulsion. [41]. While the zeta potential first increased and then decreased with the increase in BBG ratio (Figure 5b) (*p* < 0.05), the highest zeta potential of emulsion reached −16.55 ± 0.55 mV at BBG:SCMC = 9:1. This may be due to electrostatic interactions between proteins and polysaccharides in different ratios [43]. Studies have shown that when two biopolymers have opposite charges, the complex formation is mainly through electrostatic interactions [53]. Therefore, BBG/SCMC formation can be attributed to the occurrence of electrostatic interactions between the two. Meanwhile, the zeta potentials of BBG/SCMC–CE and BBG/SCMC–NE did not change significantly (Figure 5c) with the increase in polyphenol content, suggesting that the surface charge of the emulsion droplets was almost unaffected by the added polyphenols during the binding process, indicating that the polyphenols were mainly distributed in the hydrophobic interior of the proteins instead of the surface [31]. While the zeta potential of the emulsions was closely related to the magnetic stirring time, the zeta potentials of BBG/SCMC–CE and BBG/SCMC–NE first gradually increased and then tended to equilibrate with the extension of the magnetic stirring time (Figure 5d). This may be due to the fact that with the increase in magnetic stirring time, the active substances in the oil phase were oxidized and changed their properties, which increased the zeta potential of the emulsion [54]. As the magnetic stirring time increases, the active substances are completely destroyed, and the zeta potential no longer changes.

### 3.2. Response Surface Experiment Optimization

The above results fully analyzed the effects of different treatments on emulsions, where the effect on emulsion particle size and zeta potential was highly significant (*p* < 0.05). In order to further optimize the optimal preparation process of the emulsion, the Box–Behnken experimental design principle was used to select the total solids content of 1%, 2%, and 3%; the substrate ratios of 10:0, 9:1, and 8:2; the polyphenol contents of 0.1%, 0.2%, and 0.3%; and the magnetic stirring time of 50 min, 60 min, and 70 min. The combined value of particle size and zeta potential were selected as the response surface values for the next optimization step of the response surface experiments.

#### 3.2.1. Response Surface Optimization Test Results and Analysis of Variance

Based on the results in Table 2, the data were analyzed by multiple regression fitting via Design–Expert 13.0 software, and quadratic multinomial equations were obtained using total solids content A, proportion of substrate B, concentration of polyphenols C, and magnetic churning time D as independent variables and with the combined value of particle size (by weight 40%) and zeta potential (by weight 60%) Y as the dependent variable: Y = 0.982 + 0.0158A + 0.0225B + 0.0117C + 0.0133D + 0.005AB − 0.005AC − 0.0025AD − 0.0025BD − 0.0956A^2^ − 0.0706B^2^ − 0.0543C^2^ − 0.0618D^2^, R^2^ = 0.9535. The calibration coefficient R^2^Adj = 0.9070 indicates that 95.35% of the variation in response values can be explained by the model, and 90.70% of test results are influenced by test factors. The actual test results were analyzed by ANOVA using the regression equation, and the results are shown in Table 3.

As can be seen in Table 3, the model is highly significant (*p* < 0.0001), and the misfit term is not significant (*p* = 0.2371 > 0.05), indicating that the regression model is valid. The effect of the independent variable B on the composite value Y is highly significant (*p* < 0.01), and the effects of A, C, and D on Y are significant (*p* < 0.05); the interaction terms AB and AD in the model have highly significant effects on the composite value Y (*p* < 0.01), and the effects of AB, AC, AD, BC, BD, and CD are not significant (*p* > 0.05). The effects of the secondary terms A^2^, B^2^, C^2^, and D^2^ in the model on the composite value Y all reached the highly significant level (*p* < 0.01). In summary, the degree of influence of each factor on the Y composite value of elasticity and sensory scores was B > A > D > C, i.e., proportion of substrate > total solids content > magnetic stirring time > polyphenol concentration.

#### 3.2.2. Analysis of Interactions between Factors

**Figure 6:**
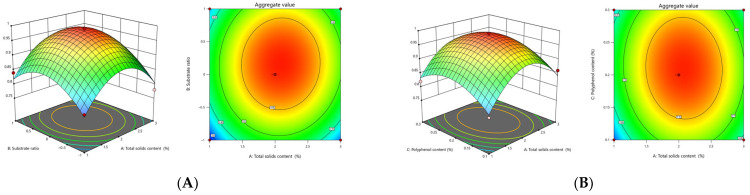

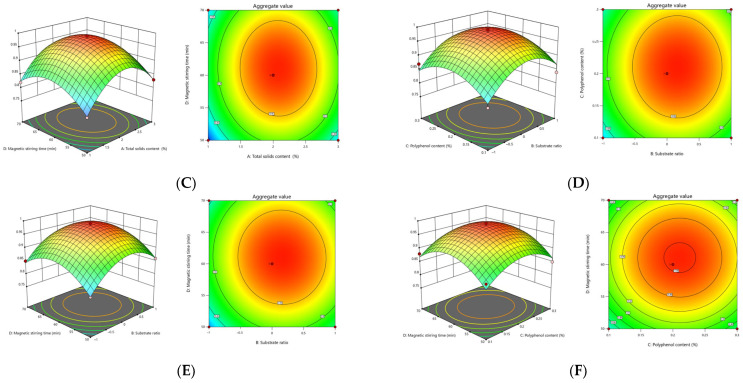
Effect of factor interactions on composite values. (**A**) Interaction of total solids content with percentage of substrate. (**B**) Interaction of total solids content with active substance concentration. (**C**) Interaction of total solids content with magnetic stirring time. (**D**) Interaction between substrate ratio and active substance concentration. (**E**) Interaction between substrate ratio and magnetic stirring time. (**F**) Interaction between active substance concentration and magnetic stirring time.

In order to more intuitively reflect the effects of interactions between the four factors of total solids content (A), proportion of substrate (B), polyphenol addition (C), and magnetic stirring time (D) on the composite value Y, response surfaces and contour plots of the relationship between each of the two factors and the composite value were plotted using Design–Expert 13.0 software (Figure 6). The closer the contour shape of the response surface is to an ellipse, the steeper the surface is, indicating that the interaction between independent variables has a more significant effect on the response surface value. As can be seen in Figure 6**,** the contours of the interaction terms AB, AC, AD, BC, and BD are elliptical and densely distributed with steeper surfaces, indicating that the interactions between total solids content and the proportion of substrate, polyphenol content, and magnetic churning time are significant, and the effects on response surfaces are more pronounced, which is consistent with the results in Table 3.

#### 3.2.3. Verification Experiment

**Figure 7:**
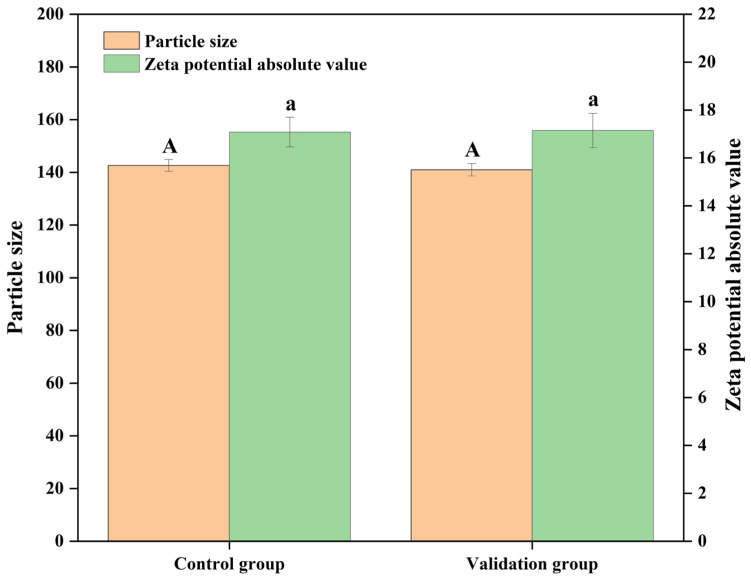
Verification experiment results. Uppercase letters indicate significant (*p* < 0.05) differences in particle size between treatment groups, and lowercase letters indicate significant (*p* < 0.05) differences in absolute value of zeta potential between treatment groups.

Through the regression model prediction, the optimal preparation process of BBG/SCMC emulsion prepared by ultrasonic technology was determined as follows: total solids content of 2.083%, substrate ratio of 1.3867:8.6133, polyphenol content of 0.21%, and magnetic stirring time of 61.028 min. At this time, the particle size and zeta potential of the nanoemulsion were 133.705 nm and −16.949 mV, respectively. Considering the possibility of practical operation, the optimal process was adjusted to 2% total solids, 9:1 substrate ratio, 0.2% polyphenol content, and magnetic stirring time of 60 min. As shown in Figure 7, and the validation was repeated three times, which made the actual value and theoretical value of the model close to each other and showed that this model was not suitable for the optimization of BBG/SCMC. It has some feasibility in optimizing the preparation process of BBG/SCMC nanoemulsion.

### 3.3. Structural Characterization of BBG/SCMC

#### 3.3.1. Surface Morphology Analysis (SEM)

**Figure 8:**
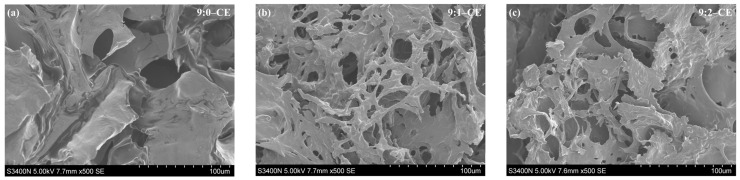

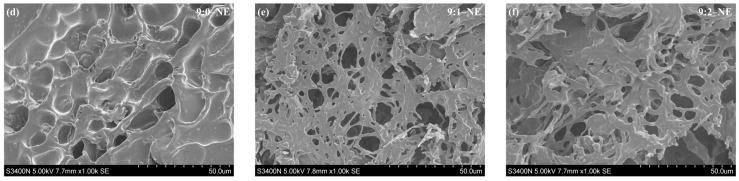
Scanning electron microscope (SEM) images of different ratios BBG/SCMC–CE (**a**–**c**) and BBG/CMC–NE (**d**–**f**) (500×; 1000×).

For the purpose of micro-morphological characterization of polyphenol–BBG/SCMC covalent complexes, it was observed using electron scanning electron microscope, as shown in Figure 8. The SEM results showed that the surface of BBG/SCMC–CE was rough, with varying pore sizes and the presence of lamellar aggregates. On the other hand, the surface of BBG/SCMC–NE was flatter and more regular, with tighter cross-linking and a dense mesh structure. This is similar to the microstructure of films prepared by Picchio et al. [55] with casein cross-linked tannins (TA) and food-grade bovine bone gelatin nanoparticles prepared by Gong et al. [56]. The microstructure of BBG/SCMC–NE was more compact relative to that of BBG/SCMC–CE, and the change in its microstructure could be attributed to the covalent reaction of polyphenols with proteins due to ultrasound, which led to the change in chemical bonding, thus altering the microstructure and making the polyphenols more stable after ultrasound when they were combined with BBG/SCMC.

#### 3.3.2. Fourier Infrared Spectral Analysis (FTIR)

**Figure 9:**
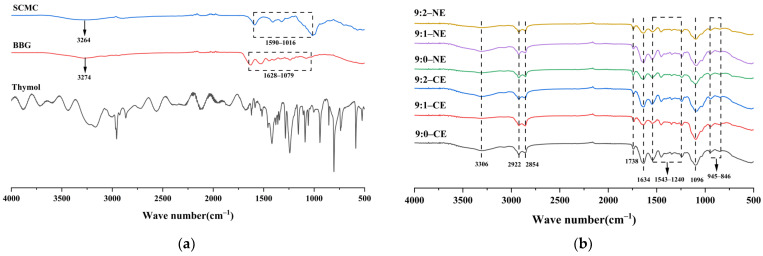
FTIR analysis of BBG, SCMC, and THY (**a**). FTIR analysis plots of BBG/SCMC–CE and BBG/SCMC–NE with different ratios (**b**).

As shown in Figure 9a,b, FTIR spectroscopy is an important technique that can provide the ability to analyze the secondary structure of emulsions and the nature of interactions within nanocomplexes [31]. The FTIR spectra of BBG/SCMC emulsions (Figure 9) showed the presence of five absorption bands in the range of 400–4000 cm^−1^, namely amide A, amide B, amide I, amide II, and amide III bands [57]. The characteristic absorption peak of 3500–3250 cm^−1^ is the amide A band, which is mainly due to the –OH stretching vibration, and the reason for this can be explained by the intermolecular interaction between the hydroxyl group in SCMC and the carboxyl group in gelatin (Figure 9a) [3]. The characteristic absorption peak at 2950–2850 cm^−1^ is the amide B band, which is mainly due to the antisymmetric stretching vibration of the C–H bond [4]. The characteristic absorption peak at 1750–1600 cm^−1^ is the amide I band, which is caused by the stretching vibration of the carbonyl group (C=O) in the addictive amine bond along the backbone of the gelatin molecular chain [58]. The characteristic absorption peak at 1550–1300 cm^−1^ is the amide II band, which is mainly caused by the bending vibration of the N–H bond in conjunction with the stretching vibration of the C–N bond [59]. The characteristic absorption peak at 1250 cm^−1^ is the amide III band, which is mainly caused by the combination of the rocking vibration of the glycine backbone and the proline side chain–CH_2_ group in the gelatin molecular chain, the stretching vibration of the C–N bond, and the bending vibration of the N–H bond [60]. Vibration in the band at 1000–840 cm^−1^ may be due to the presence of polyphenols, which alter the intensity of gelatin absorption peaks [3]. Both BBG/SCMC emulsions showed similar FTIR spectra with an increasing percentage of bovine bone gelatin. Comparing the peak shapes of gelatin and composite solution, it can be found that the wave numbers corresponding to the bending vibration peaks did not change significantly during the composite process and ultrasonication. As the proportion of bovine bone gelatin increased, the distance between BBG/SCMC and polyphenols increased, and the degree of cross-linking decreased, leading to a decrease in the intensity of absorption peaks. In addition, the intensity of the characteristic peaks of thymol in the emulsion weakened or even disappeared at 1241 cm^−1^ and 587 cm^−1^, suggesting that there was a strong physical cross-linking between thymol and the wall material, which embedded thymol molecules in protein–polysaccharide hydrophobic cavities, thereby reducing thymol molecule movement [61]. And it can be obtained from Figure 9b that there was a slight wave number shift between emulsions and nanoemulsion with different substrate ratios at different absorption peak intensities, indicating that a mixed-phase system is formed between different ratios of BBGs and SCMCs and that there is a slight difference between the main groups obtained by their reactions [62].

#### 3.3.3. X-ray Diffraction Analysis (XRD)

**Figure 10:**
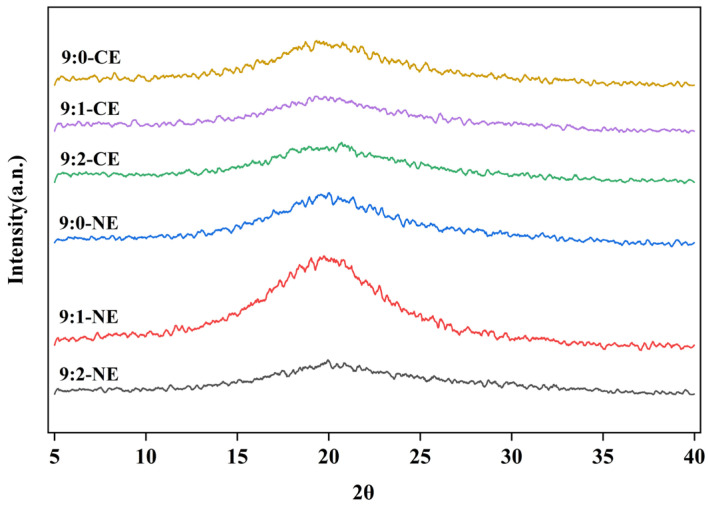
X-ray diffraction analysis plots of BBG/SCMC–CE and BBG/SCMC–NE with different ratios.

To further confirm the effect of protein and polysaccharide complexation on the molecular chain structure of gelatin, crystal and amorphous structures were analyzed using XRD on BBG/SCMC–CE and BBG/SCMC–NE. As shown in Figure 10, the diffraction peaks existed only at 2θ ≈ 20° with the change of the proportion of bovine bone gelatin. In general, hydrophobic bioactives are released more efficiently from the amorphous state than from the crystalline state [63]. According to the previous results of Soltanzadeh et al. [6], both pure gelatin-based films and gelatin/watercress seed gum composite films showed characteristic peaks near 2θ = 20°. Observations in this study confirmed that characteristic peaks of polyphenols were not observed due to encapsulation of polyphenols by BBG/SCMC emulsions, indicating that polyphenol infiltration did not have a significant effect on the crystallinity of the emulsions [64,65]. This is consistent with SEM (Figure 8) and FTIR (Figure 9) results. And the intensity of the diffraction peaks of the nanoemulsion changed significantly with the change in the ratio of BBG and SCMC, which can be explained by the different molecular weights of different ratios of BBG and SCMC aggregated during the complexation process [3]. In addition, XRD analysis confirmed the DSC results that BBG/SCMC polyphenol complexes existed in compact form in nanocomplexes.

### 3.4. Thermal Stability and Oxidation Resistance of BBG/SCMC

#### 3.4.1. Differential Scanning Calorimeter Analysis (DSC)

**Figure 11:**
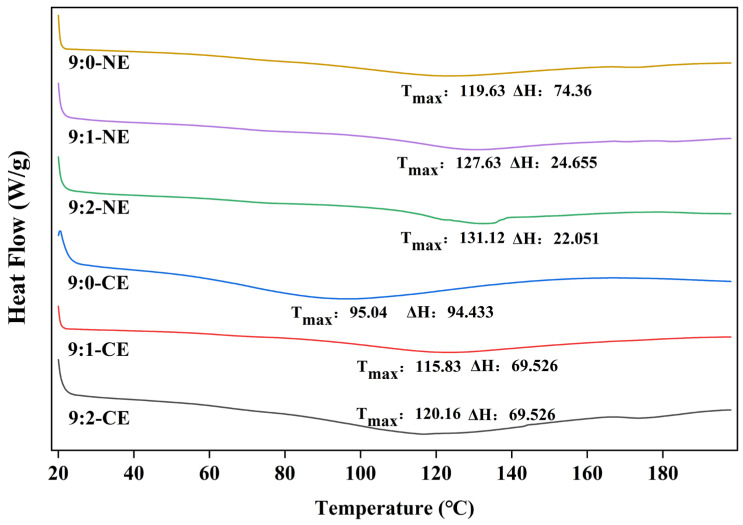
Differential scanning calorimetry (DSC) plots of BBG/SCMC–CE and BBG/SCMC–NE with different ratios.

Differential scanning calorimetry (DSC), which typically characterizes complexes by comparing the thermal behavior of individual components and complexes, is able to indicate the melt transition temperature (T_max_) and enthalpy of melting (ΔH) of gelatin-based emulsions [66]. The DSC results (Figure 11) showed that many thermal transformation behaviors occurred when BBG/SCMC emulsions were heated. The T_max_ of BBG/SCMC–CE and BBG/SCMC–NE with different substrate ratios increases with the increase in SCMC ratio. In general, an increase in thermal denaturation temperature corresponds to an increase in complex stability [67]. Thus, complexing with polysaccharides can improve the thermal stability of proteins, which is consistent with the effect of using different amounts of corn starch polysaccharides on the thermal stability of peanut isolate proteins, as studied by Han et al. [67], and the effect of gum Arabic (GA) or carboxymethyl cellulose (CMC) on the thermal stability of potato protein (PP) thermal stability with consistent results, as studied by Stounbjerg et al. [68]. Figure 11 shows the higher peak temperature of BBG/CMC–NE relative to BBG/CMC–CE, which may be related to the change in conformation due to the shear force exerted under ultrasound after sonication [69]. In addition, the lower enthalpy of the nanoemulsion after sonication may be due to the fact that the nanoemulsion requires a lower enthalpy to disrupt interchain interactions due to the cavitation effect of sonication.

#### 3.4.2. DPPH Free Radical Scavenging Capacity

**Figure 12:**
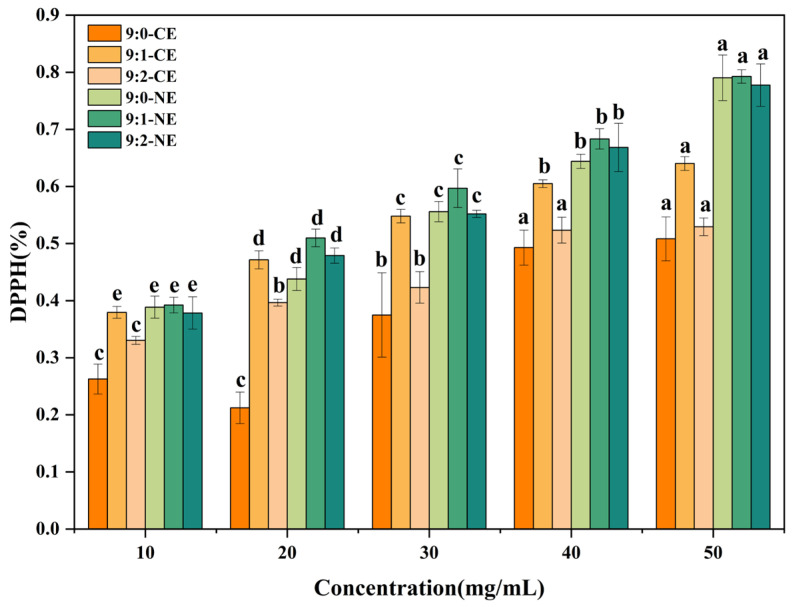
DPPH radical scavenging capacity of BBG/SCMC–CE and BBG/SCMC–NE with different ratios. Different lowercase letters indicate significant differences between different levels of the factor (*p* < 0.05).

The DPPH molecule is a stabilized synthetic free radical, and the antioxidant activity of a substance is usually measured by the scavenging capacity of the DPPH radical [70]. Regarding the DPPH radical scavenging ability of BBG/SCMC–CE and BBG/SCMC–NE, as shown in Figure 12, the DPPH radical scavenging activity increased gradually with increasing concentration, and the DPPH radical scavenging rate of 9:1 BBG/SCMC–NE was up to 79.25%. In general, the presence of a large number of hydroxyl groups in the phenolic structure is considered to have considerable antioxidant activity [71]. The antioxidant activities of both crude and nanoemulsion with different BBG and SCMC ratios in this study were different, which can be attributed to the fact that polysaccharides complexed with proteins formed a stable complex network, and most polyphenols were not destroyed during the complexing process; they were completely encapsulated by protein–polysaccharide complexes in their internal structure, which gave complexes a strong antioxidant capacity [72]. It can be concluded that the antioxidant activity of the covalent compound is higher than that of natural gelatin.

### 3.5. Encapsulation Efficiency and Release Performance

**Figure 13:**
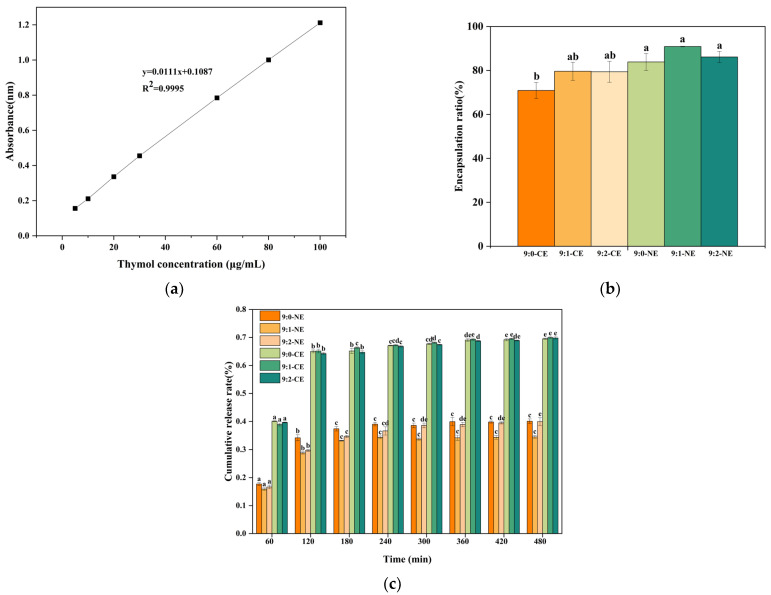
Encapsulation efficiency and release properties of BBG/SCMC–CE and BBG/SCMC–NE with different ratios. (**a**) The standard graph of thymol. (**b**) The encapsulation efficiency of BBG/SCMC–CE and BBG/SCMC–NE calculated from the standard curve. (**c**) The release performance of BBG/SCMC–CE and BBG/SCMC–NE at different times. Different lowercase letters indicate significant differences between different levels of the factor (*p* < 0.05).

The encapsulation rate is a key index to characterize the amount of thymol encapsulated in a nanoemulsion. The encapsulation efficiency and release properties of BBG/SCMC–CE and BBG/SCMC–NE are shown in Figure 13. The linear regression equation of the standard curve of thymol was tested as y = 0.0111x + 0.1087, R^2^ = 0.9995, with a good linear relationship (Figure 13a), and then, combined with Equation (2) it was calculated that the encapsulation rates of BBG/SCMC–CE prepared were 70.88, 79.60, and 79.39%, respectively. BBG/SCMC–NE encapsulation rates were 83.85, 90.88, and 86.13%, respectively (Figure 13b). In this study, covalently bound protein–polysaccharide nanoemulsion were prepared by ultrasonic technology to obtain “inner hydrophobic and outer hydrophilic” pore structures, which can encapsulate hydrophobic substances in the inner cavity. Shown in Figure 13b, the encapsulation efficiency of 9:1 BBG/SCMC–NE was up to 90.88%. In addition, the protein–polysaccharide covalent composite nanoemulsion prepared in this study showed more impressive thymol encapsulation compared to chitosan nanoemulsion containing thymol prepared by Liu and Liu [32].

The thymol-release atmosphere in BBG/SCMC–CE and BBG/SCMC–NE has three stages, which are sudden release, slow increase, and leveling off [33]. As shown in Figure 13c, both BBG/SCMC–CE and BBG/SCMC–NE exhibited sudden release up to 120 min, with cumulative release rates of 65.00, 65.15, 64.25, 34.22, 28.81, and 29.72%, respectively. The cumulative release rate increased slowly during 120–180 min, which was attributed to the slow release of thymol from the emulsion. The cumulative release rate gradually equilibrated in 180–480 min. This is similar to the trend of active substance release in corn alkyd protein nanofilms prepared by Jiang et al. [33] and starch/poly (vinyl alcohol) antimicrobial active films prepared by Qiao et al. [73]. The high release of thymol in emulsions during the initial phase inhibits bacteria in the delayed and logarithmic phases at the onset of proliferation and, during the sustained release phase, inhibits a small percentage of bacteria that still grow and multiply during the stabilization and decay phases, giving emulsions long-lasting antimicrobial effects [74]. The release rate of BBG/SCMC–NE was significantly lower than that of BBG/SCMC–CE (*p* < 0.05), which may be due to the fact that the nanoemulsion prepared by ultrasound had denser and finer pores (Figure 8), which resulted in a lower release rate of thymol and acted as a retardation of release.

## 4. Conclusions

In this paper, bovine bone gelatin/sodium carboxymethyl cellulose nanoemulsion was prepared, the process was optimized to obtain the optimum process, and its structure, thermal stability, and antioxidant properties were investigated. The optimum process was found to be 2% total solids, 9:1 substrate ratio, 0.2% polyphenol content, and magnetic stirring time of 60 min. The SEM results showed that the 9:1 nanoemulsion (9:1–NE) showed a denser and more homogeneous reticulated structure. The FTIR results showed that the cross-linking degree of BBG/SCMC decreased with the increase in the proportion of bovine bone gelatin, resulting in a decrease in peak absorption intensity. The XRD results showed that peak intensity decreased with the increase in the proportion of bovine bone gelatin, and gelatin was more closely associated with polysaccharides and polyphenols. In addition, 9:1–NE has higher thermal stability, radical scavenging of DPPH, encapsulation efficiency, and slow release properties. This leads to the conclusion that BBG/SCMC–NE with a substrate ratio of 9:1 is the best sample. Therefore, BBG/SCMC–NE loaded with polyphenol has great potential as a new biodegradable packaging material for the application of active packaging, thus opening up new prospects for preventing food spoilage and improving food quality.

## Figures and Tables

**Table 1:** Response surface test factor-level table.

Level	Factors			
	*A* Total Solids Content/%	*B* Concentration of Active Material/%	*C* Base Material Ratio	*D* Magnetic Stirring Time/min
−1	1	0.1	10:0	50
0	2	0.2	9:1	60
1	3	0.3	8:2	70

**Table 2:** Response surface test design scheme and results.

Number	*A*	*B*	*C*	*D*	Y_1_ (*Particle Size*)	Y_2_ (*Zeta Potential*)	Y (*Aggregate Value*)
1	−1	−1	0	0	147	−8.93	0.79
2	1	−1	0	0	187	−9.66	0.78
3	−1	1	0	0	149	−12.68	0.84
4	1	1	0	0	190	−13.24	0.85
5	0	0	−1	−1	142	−16.52	0.86
6	0	0	1	−1	148	−16.77	0.85
7	0	0	−1	1	146	−16.83	0.88
8	0	0	1	1	144	−17.02	0.87
9	−1	0	0	−1	153	−16.31	0.79
10	1	0	0	−1	189	−16.72	0.83
11	−1	0	0	1	156	−16.57	0.82
12	1	0	0	1	194	−16.89	0.85
13	0	−1	−1	0	152	−9.47	0.81
14	0	1	−1	0	155	−12.55	0.84
15	0	−1	1	0	161	−9.83	0.87
16	0	1	1	0	164	−12.68	0.9
17	−1	0	−1	0	135	−16.56	0.79
18	1	0	−1	0	152	−16.74	0.86
19	−1	0	1	0	138	−16.83	0.82
20	1	0	1	0	155	−16.92	0.87
21	0	−1	0	−1	147	−9.23	0.81
22	0	1	0	−1	158	−12.57	0.86
23	0	−1	0	1	142	−9.66	0.85
24	0	1	0	1	153	−13.21	0.89
25	0	0	0	0	142	−17.08	0.98
26	0	0	0	0	143	−17.08	0.98
27	0	0	0	0	143	−17.09	0.99
28	0	0	0	0	143	−17.09	0.98
29	0	0	0	0	142	−17.09	0.98

**Table 3:** Analysis of variance in response surface test.

Source	Sum of Squares	D.F.	M.S.	F-Value	*p*-Value	Sig.
Model	0.0920	4	0.0066	17.57	<0.0001	**
A	0.0030	1	0.0030	8.04	0.0132	*
B	0.0061	1	0.0061	16.24	0.0012	**
C	0.0016	1	0.0016	4.37	0.0554	*
D	0.0021	1	0.0021	5.70	0.0316	*
AB	0.0001	1	0.0001	0.2673	0.6132	
AC	0.0001	1	0.0001	0.2673	0.6132	
AD	0.0000	1	0.0000	0.0668	0.7998	
BC	0.0000	1	0.0000	0.0000	1.0000	
BD	0.0000	1	0.0000	0.0668	0.7998	
CD	1.388 × 10^−17^	1	1.388 × 10^−17^	3.709 × 10^−14^	1.0000	
A^2^	0.0532	1	0.0532	142.25	<0.0001	**
B^2^	0.0279	1	0.0279	74.56	<0.0001	**
C^2^	0.0158	1	0.0158	42.19	<0.0001	**
D^2^	0.0210	1	0.0210	56.00	<0.0001	**
Residual	0.0052	4	0.0004			
Lack of Fit	0.0050	0	0.0005	7.08	0.2371	
Pure Error	0.0003	4	0.0001			
Cor Total	0.0973	8				

Note: A, total solids content; B, proportion of substrate; C, concentration of active substance; D, magnetic stirring time; D.F., degree of freedom; M.S., mean square; Sig., significance. * significant difference (*p* < 0.05); ** highly significant difference (*p* < 0.01).

## Data Availability

The original contributions presented in the study are included in the article, further inquiries can be directed to the corresponding author.
